# Association between Dietary Patterns and the Indicators of Obesity among Chinese: A Cross-Sectional Study

**DOI:** 10.3390/nu7095376

**Published:** 2015-09-17

**Authors:** Long Shu, Pei-Fen Zheng, Xiao-Yan Zhang, Cai-Juan Si, Xiao-Long Yu, Wei Gao, Lun Zhang, Dan Liao

**Affiliations:** 1 Department of Nutrition, Zhejiang Hospital, Xihu district, Hangzhou 310013, Zhejiang, China; E-Mails: shulong19880920@126.com (L.S.); kuaidou09@163.com (P.-F.Z.); xiaosi_32075001@126.com (C.-J.S.); xly2008hi@163.com (X.-L.Y.); gaowei05715133@163.com (W.G.); zhanglun306@163.com (L.Z.); liaodan0203@sina.com (D.L.); 2 Department of Digestion, Zhejiang Hospital, Xihu district, Hangzhou 310013, Zhejiang, China

**Keywords:** dietary patterns, obesity, China, cross-sectional study, factor analysis

## Abstract

No previous study has investigated dietary pattern in association with obesity risk in a middle-aged Chinese population. The purpose of this study was to evaluate the associations between dietary patterns and the risk of obesity in the city of Hangzhou, the capital of Zhejiang Province, east China. In this cross-sectional study of 2560 subjects aged 45–60 years, dietary intakes were evaluated using a semi-quantitative food frequency questionnaire (FFQ). All anthropometric measurements were obtained using standardized procedures. The partial correlation analysis was performed to assess the associations between dietary patterns and body mass index (BMI), waist circumference (WC), and waist to hip ratio (WHR). Multivariate logistic regression analysis was used to examine the associations between dietary patterns and obesity, with adjustment for potential confounders. Four major dietary patterns were extracted by means of factor analysis: animal food, traditional Chinese, western fast-food, and high-salt patterns. The animal food pattern was positively associated with BMI (*r* = 0.082, 0.144, respectively, *p* < 0.05) and WC (*r* = 0.102, 0.132, respectively, *p* < 0.01), and the traditional Chinese pattern was inversely associated with BMI (*r* = −0.047, −0.116, respectively, *p* < 0.05) and WC (*r* = −0.067, −0.113, respectively, *p* < 0.05) in both genders. After controlling for potential confounders, subjects in the highest quartile of animal food pattern scores had a greater odds ratio for abdominal obesity (odds ratio (OR) = 1.67; 95% confidence interval (CI): 1.188–2.340; *p* < 0.01), in comparison to those from the lowest quartile. Compared with the lowest quartile of the traditional Chinese pattern, the highest quartile had a lower odds ratio for abdominal obesity (OR = 0.63; 95% CI: 0.441–0.901, *p* < 0.05). Conclusions: Our findings indicated that the animal food pattern was associated with a higher risk of abdominal obesity, while the traditional Chinese pattern was associated with a lower risk of abdominal obesity. Further prospective studies are warranted to confirm these findings.

## 1. Introduction

Obesity is a health risk factor for chronic diseases such as cardiovascular diseases, type 2 diabetes, hypertension and several types of cancers [1], and has become a major concern in all populations worldwide [2]. According to the World Health Organization (WHO) statistics, 39% of adults aged 18 years and over were overweight in 2014, and 13% were obese [3]. In China, the prevalence of obesity in Chinese adults has increased dramatically from 7.1% in 2002 to approximately 12.0% in 2010 [4]. It is well known that obesity is considered a complex multifactorial chronic disease that may be associated with some factors, including genetic and environmental factors, and especially dietary factors [5].

Over the past several decades, some previous epidemiological studies specifically focused on diet modification as an important role for the prevention of obesity, and they have reported the associations between the intakes of individual nutrients or foods and food groups and the risk of obesity [6,7]. Nonetheless, due to the complexity of diets and the potential interactions between food components [8], these analyses revealed the limited impact of diets on the occurrence of obesity. Consequently, the analysis of dietary patterns has been increasingly used in nutritional epidemiology. Furthermore, because of its ability to examine the holistic effect of diet, dietary pattern analysis also has been used to determine the associations between diet and some chronic diseases [9,10].

More recently, due to rapid economic, social, and cultural changes in China, the dietary pattern is shifting from the traditional pattern with a high consumption of staple food, coarse grains, vegetables, and fruits, to the western pattern with a high consumption of animal foods and other high energy-dense foods [11]. Following the dietary transition, chronic diseases (e.g., obesity, hypertension) are accelerating in China [12]. Therefore, it is important to examine the dietary pattern and its association with obesity in the Chinese population, especially in the middle-aged and elderly population, who are more prone to be influenced by some non-communicable chronic diseases. In fact, the relationship between dietary patterns and obesity has scarcely been reported in the Chinese population [13,14]. To the best of our knowledge, no previous study has reported the relationship between dietary patterns and obesity in Chinese adults aged 45–60 years. Therefore, the aim of the study was to identify major dietary patterns among a Chinese population aged 45–60 years, and evaluate the associations of these patterns with the indicators of obesity.

## 2. Subjects and Methods

### 2.1. Study Population

During the period of July 2014 through June 2015, this cross-sectional study was carried out in Hangzhou, the capital of Zhejiang Province, east China. The study sample was taken from eight areas (Gongshu, Shangcheng, Xiacheng, Jianggan, Xihu, Bingjiang, Xiaoshan, and Yuhang) and five counties (Fuyang, Tonglu, Chunan, Jiande, and Linan) by a stratified cluster random-sampling method. We chose one residential village or community from every county or area randomly, according to resident health records, with participants aged between 45 and 60 years residing in the selected villages or communities. A total of 2734 eligible participants (1440 male, 1294 female) were invited to attend a health examination at the Medical Center for Physical Examination, Zhejiang Hospital, where the participant was face-to-face interviewed by a trained interviewer using written questionnaires. We excluded an additional 36 participants because of incomplete anthropometric information, as well as 138 participants who provided missing or incomplete information on their dietary intake. Finally, 2560 participants were included in our analyses. The protocol of this study was approved by the institutional review and ethics committee of Zhejiang Hospital, Zhejiang Province, China, and written informed consent was obtained from all participants.

### 2.2. Assessment of Dietary Intake

The 58 food items in the semi-quantitative food frequency questionnaire (FFQ) were divided into 33 food groups based on the roles of food in diet and nutritional characteristics (Table S1). This FFQ was based on the food frequency questionnaire used in the 2010 China National Nutrition and Health Survey (CNNHS). Participants were asked to recall the frequency of each food item over the previous 12 months and the estimated portion size, using local weight units (1 Liang = 50 g) or natural units (cups). The frequency of food intake was measured using nine categories: never, <12 times/year, 1–3 times/month, 1–2 times/week, 3–4 times/week, 5–6 times/week, 1 time/day, 2 times/day, and 3 times/day. The selected frequency category for each food item was converted into a daily intake.

### 2.3. Identification of Dietary Patterns

The Kaiser-Meyer-Olkin Measure of Sample Adequacy and the Bartlett Test of Sphericity were used to assess data adequacy for factor analysis. We used the factor analysis (principal component) to identify the dietary patterns. The factors were rotated by orthogonal transformation (varimax rotation) to maintain the uncorrelated nature of the factors and greater interpretability; the eigenvalue and scree plot were applied to decide which factors remained [15]. After evaluating the eigenvalues, the scree plot test, and interpretability, eigenvalues ≥2.0 were retained. Factor groups with a factor loading ≥|0.4| were considered to significantly contribute to the pattern in this study. The labeling of dietary patterns was based on the interpretation of foods with high factor loadings for each dietary pattern [16]. According to these criteria, four major dietary patterns were identified. The animal food pattern was characterized by high intakes of rice, mushroom, red meat, fish and shrimp, seafood, and fats/oils. The traditional Chinese pattern was characterized by high intakes of rice, steamed bun/noodles, coarse grains, tubers, fresh vegetables and fruits, fish and shrimp, miscellaneous beans and tea. The western fast-food pattern was characterized by high intakes of fast foods, snacks, chocolates, coffee, and drinks. The high-salt pattern was characterized by high intakes of pickled vegetables, processed and cooked meat, bacon and salted fish and bean sauce.

### 2.4. Assessment of Anthropometric Measurements

Height was measured to the nearest 0.1 cm with subjects standing without shoes. Body weight in light clothes was measured to the nearest 0.1 kg using a digital scale. Body mass index (BMI) was calculated as weight in kilograms divided by squared height in meters. waist circumference (WC) was measured halfway between the lower rib edge and the upper iliac crest by means of a metric measure with an accuracy of 1 mm [17] and hip circumstance was measured at the maximum level over light clothing by using an inelastic plastic tape [18]. All measurements were performed by nurses trained to use standardized procedures.

### 2.5. Assessment of Other Variables

The validity of the questionnaire in assessing physical activity was described elsewhere [19]. The physical activity levels were expressed as metabolic equivalents in hours per week (MET-h/week). Information on smoking status included the categories of never smokers, current smokers, and former smokers. The educational level was categorized in three classes: primary school or below, middle and high school, junior college or above. Total energy intake was estimated through the FFQ, expressed in kilocalorie per day (kcal/day) and categorized according to quartile.

### 2.6. Definition of Terms

Obesity was defined by BMI ≥ 28 kg/m^2^ and abdominal obesity was defined as (male: WC ≥ 85 cm; female: WC ≥ 80 cm) in a Chinese population [20].

### 2.7. Statistical Analyses

Factor scores were categorized into quartiles (quartile1 represented a low consumption of this food pattern while quartile 4 represented a high consumption of this food pattern). The total participants numbered 2560 in our analyses. Thus, the number of participants in each quartile of each dietary pattern is 640. The characteristics of the study participants were calculated across quartiles of each dietary pattern. Data are presented as the mean ± standard deviation (SD) for continuous variables and as a sum (percentages) for categorical variables. We used analysis of variance (ANOVA) to describe mean differences by continuous variables and the chi-squared test to evaluate the difference between categorical variables. Analysis of covariance was used to compare the difference of BMI, WC, and waist to hip ratio (WHR) in the highest categories compared with the lowest categories of different dietary patterns. The partial correlation analysis was performed to assess the associations between dietary patterns and BMI, WC, and WHR. Multivariate logistic regression analysis was used to examine the associations between dietary patterns and obesity, with adjustment for age, smoking status, economic income, educational level, physical activity level, and total energy intake.

Statistical analyses were performed using the SPSS software package version 16.0 for Windows (SPSS Inc., Chicago, IL, USA). Two-sided *p*-values < 0.05 were considered statistically significant.

## 3. Results

Both the Kaiser-Meyer-Olkin index (0.798) and Bartlett**^’^**s test (*p* < 0.001) showed that the correlation among the variables was sufficiently strong for a factor analysis [21]. Four major dietary patterns were extracted by means of the factor analysis: animal food, traditional Chinese, western fast-food, and high-salt patterns. These factors explained 27.9% of the whole variance. In addition, the factor-loading matrixes for these dietary patterns were shown in Table 1.

**Table 1:** Factor-loading matrix for major dietary patterns among 2560 Chinese adults aged 45–60 years *.

Food Groups	Dietary Patterns			
	Animal Food	Traditional Chinese	Western Fast-Food	High-Salt
Rice	0.476	0.560	-	-
Steamed bun/noodles	-	0.411	-	-
Coarse grains	-	0.544	-	-
Tubers	-	0.535	-	-
Fresh vegetables and fruits	-	0.540	-	-
Pickled vegetables	-	-	-	0.561
Mushroom	0.462	-	-	-
Red meat	0.650	-	-	-
Processed and cooked meat	-	-	-	0.517
Fish and shrimp	0.599	0.530	-	-
Seafood	0.568	-	-	-
Bacon and salted fish	-	-	-	0.601
Miscellaneous bean	-	0.535	-	-
Bean sauce	-	-	-	0.436
Fats/oils	0.402	-	-	-
Fast foods	-	-	0.467	-
Snacks	-	-	0.456	-
Chocolates	-	-	0.485	-
Coffee	-	-	0.450	-
Drinks	-	-	0.561	-
Tea	-	0.438		-
Variance of intake explained (%)	7.5	7.2	7.2	6.0

* Absolute values < 0.4 were excluded for simplicity.

The characteristics of study participants across quartile categories of the dietary pattern scores were shown in Table 2. Participants in the top quartile of the animal food pattern were more likely to be smokers, significantly younger, and had a higher prevalence of general and abdominal obesity, and higher education level, income, and physical activity. Conversely, compared with those in the lowest quartile, individuals in the highest quartile of the traditional Chinese pattern were more likely to be female, less likely to be smokers, significantly older, and had a lower prevalence of general and abdominal obesity and hypertension and higher economic income. In addition, participants in the highest quartile of the western fast-food pattern were more likely to be female, significantly younger, and had higher economic income than those in the lowest quartile. In contrast, participants in the highest quartile of the high-salt pattern were more likely to be smokers, male, and had a lower education level and economic income than those in the lowest quartile.

**Table 2:** Characteristics of the study participants by quartile (Q) categories of dietary pattern scores in the Hangzhou.

	Animal Food		*p	Traditional Chinese		*p	Western Fast-Food		*p	High-Salt		*p
	Q1 (*n* = 640)	Q4 (*n* = 640)		Q1 (*n* = 640)	Q4 (*n* = 640)		Q1 (*n* = 640)	Q4 (*n* = 640)		Q1 (*n* = 640)	Q4 (*n* = 640)	
Age (year)	51.8 ± 0.3	50.3 ± 0.2	**<0.001**	50.0 ± 0.2	51.9 ± 0.3	**<0.001**	51.5 ± 0.2	49.7 ± 0.2	**<0.001**	50.7 ± 0.2	51.0 ± 0.2	0.789
Gender (%)			**<0.001**			**<0.001**			**<0.001**			**<0.001**
Male	328(51.2)	454(70.9)		543(84.8)	208(32.5)		433(67.6)	336(52.5)		323(50.5)	413(64.5)	
Female	312(48.8)	186(29.1)		97(15.2)	432(67.5)		207(32.4)	304(47.5)		317(49.5)	227(35.5)	
Obesity (%)	65(10.1)	108(16.8)	**<0.001**	113(17.6)	53(8.3)	**<0.001**	99(15.5)	68(10.7)	**0.01**	79(12.3)	95(14.9)	0.374
Abdominal obesity (%)	215(33.6)	258(40.3)	**0.002**	254(39.7)	211(33.0)	**0.004**	248(38.8)	229(35.8)	0.149	225(35.2)	232(36.3)	0.600
Hypertension (%)	196(30.7)	223(34.9)	0.213	261(40.8)	176(27.5)	**<0.001**	210(32.8)	162(25.3)	**0.035**	171(26.7)	215(33.6)	**0.030**
Smoking status (%)			**<0.001**			**<0.001**			0.708			**0.011**
Current	121(18.9)	253(39.5)		319(49.9)	68(10.7)		176(27.5)	184(28.8)		148(23.2)	195(30.4)	
Former	7(1.1)	11(1.7)		5(0.8)	5(0.8)		4(0.6)	6(0.9)		6(0.9)	7(1.1)	
Never	512(80.0)	376(58.8)		316(49.3)	567(88.5)		460(71.9)	450(70.3)		486(75.9)	438(68.5)	
Education level (%)			**<0.001**			0.551			0.588			**<0.001**
<High school	230(36.0)	91(14.2)		162(25.3)	143(22.4)		152(23.7)	133(20.8)		116(18.1)	177(27.7)	
High school	222(34.7)	172(26.9)		200(31.2)	196(30.7)		191(29.9)	191(29.9)		189(29.6)	203(31.7)	
>High school	188(29.3)	377(58.9)		278(43.5)	301(46.9)		297(46.4)	316(49.3)		335(52.3)	260(40.6)	
Average monthly income per person (%)			**<0.001**			**<0.001**			**<0.001**			**<0.001**
≤2000 (RMB)	261(40.8)	111(17.3)		218(34.1)	160(25.0)		207(32.4)	141(22.1)		123(19.2)	225(35.2)	
2000-3000 (RMB)	268(41.9)	244(38.1)		257(40.1)	245(38.3)		269(42.1)	219(34.2)		244(38.2)	253(39.5)	
>3000 (RMB)	111(17.3)	285(44.6)		165(25.8)	235(36.7)		164(25.5)	280(43.7)		273(42.6)	162(25.3)	
Physical activity (%)			**<0.001**			0.116			0.550			0.346
Light	452(70.7)	568(88.8)		506(79.1)	535(83.6)		517(80.8)	532(83.1)		543(84.8)	516(80.7)	
Moderate	143(22.3)	66(10.3)		106(16.6)	95(14.9)		103(16.1)	96(15.0)		76(11.9)	102(15.9)	
Vigorous	45(7.0)	6(0.9)		28(4.3)	10(1.5)		20(3.1)	12(1.9)		21(3.3)	22(3.4)	
Total energy intake (Kcal/day)	1707.1 ± 254.8	1757.6 ± 289.1	0.457	1830.6 ± 323.5	1631.6 ± 224.2	**<0.001**	1738.4 ± 269.7	1724.5 ± 311.8	0.513	1620.7 ± 224.8	1840.2 ± 320.0	**<0.001**

Categorical variables are presented as sum and percentages, and continuous variables are presented as Mean ± standard deviation (SD). * *p* values for continuous variables (analysis of variance) and for categorical variables (chi-square test). Quartiles of dietary pattern score are presented by Q1, Q2, Q3, Q4, *n* = 640. *p* < 0.05 was considered statistically significant. Monthly income per person (RMB) was presented as mean.

The difference of BMI, WC, and WHR by quartile (Q) categories of dietary pattern scores using analysis of covariance model was shown in Table 3. After controlling for gender, age, smoking status, economic income, educational level, physical activity level, and total energy intake, those participants in the highest quartile of the animal food pattern had significantly higher BMI and WC (*p* < 0.05). Conversely, participants in the highest quartile of the traditional Chinese pattern had lower BMI, WHR, and WC than those in the lowest quartile (*p* < 0.05).

After adjusting for age, smoking status, economic income, educational level, physical activity and total energy intake, partial correlation analysis indicated that: in men, the animal food pattern had a positive correlation with BMI and WC (*r* = 0.082, 0.102, respectively, *p* < 0.05); the traditional Chinese pattern was inversely associated with BMI, WC, and WHR (*r* = −0.047, −0.067, −0.062, respectively, *p* < 0.05). In women, the animal food pattern had a positive correlation with BMI (*r* = 0.144, *p* < 0.01) and WC (*r* = 0.132, *p* < 0.01); the traditional Chinese pattern was inversely associated with BMI (*r* = −0.116, *p* < 0.05) and WC (*r* = −0.113, *p* < 0.05); the high-salt pattern had a positive correlation with BMI (*r* = 0.104, *p* < 0.05). Although the coefficients of correlation were statistically significant, the association between dietary pattern score and the indicators of obesity was weak (Table 4).

**Table 3:** Analysis of covariance model to evaluate the difference of BMI, WC, and WHR by quartile (Q) categories of dietary pattern scores.

	BMI (kg/m^2^)	*p*	WHR	*p*	WC (cm)	*p*
Animal food pattern						
Q1 (*n* = 640)	24.27 ± 2.81	**0.035**	0.87 ± 0.08	0.533	84.02 ± 8.68	**0.002**
Q4 (*n* = 640)	25.10 ± 3.12		0.89 ± 0.06		87.35 ± 9.04	
Traditional Chinese pattern						
Q1 (*n* = 640)	25.13 ± 2.95	**0.023**	0.89 ± 0.06	**0.030**	87.78 ± 8.90	**<0.001**
Q4 (*n* = 640)	24.01 ± 2.76		0.86 ± 0.08		82.63 ± 8.45	
Western fast-food pattern						
Q1 (*n* = 640)	24.93 ± 3.00	0.217	0.89 ± 0.07	0.078	86.97 ± 8.58	0.193
Q4 (*n* = 640)	24.43 ± 2.93		0.87 ± 0.06		84.85 ± 8.91	
High-salt pattern						
Q1 (*n* = 640)	24.40 ± 3.11	0.259	0.87 ± 0.06	0.986	84.79 ± 9.63	0.777
Q4 (*n* = 640)	24.83 ± 2.96		0.88 ± 0.06		85.98 ± 8.61	

Adjusted for gender, age, physical activity, smoking status, economic income, educational level, and total energy intake. Abbreviation: BMI, body mass index; WHR, waist hip rate; WC, waist circumference. *p* < 0.05 was considered statistically significant; Q4: the highest quartile of dietary patterns, Q1: the lowest quartile of dietary.

**Table 4:** Partial correlation analysis for the relationship between dietary pattern score and BMI, WC, and WHR.

	BMI (kg/m^2^)	*p*	WC (cm)	*p*	WHR	*p*
Animal food pattern						
Males	0.082	**0.018**	0.102	**0.009**	0.055	0.261
Females	0.144	**0.004**	0.132	**0.008**	0.024	0.637
Traditional Chinese pattern						
Males	−0.047	**0.042**	−0.067	**0.031**	−0.062	**0.035**
Females	−0.116	**0.039**	−0.113	**0.045**	−0.007	0.826
Western fast-food pattern						
Males	−0.031	0.318	−0.022	0.344	−0.013	0.711
Females	−0.023	0.649	−0.046	0.360	−0.078	0.120
High-salt pattern						
Males	0.002	0.945	0.008	0.806	0.027	0.517
Females	0.104	**0.039**	0.024	0.632	0.019	0.807

Abbreviation: BMI, body mass index; WC, waist circumference; WHR, waist to hip ratio. Adjusted for age, smoking status, economic; income, educational level, physical activity level, and total energy intake.

The association between dietary patterns and the risk of abdominal obesity by multivariate logistic regression was shown in Table 5. After adjusting for potential confounders, subjects in the highest quartile of the animal food pattern scores had a greater odds ratio for abdominal obesity (OR = 1.67; 95% CI: 1.188–2.340; *p* < 0.01), in comparison to those from the lowest quartile; compared with the lowest quartile of the traditional Chinese pattern, the highest quartile had a lower odds ratio for abdominal obesity (OR = 0.63; 95% CI: 0.441–0.901, *p* < 0.05); Nevertheless, the western fast-food and high-salt patterns showed no association with the risk of abdominal obesity.

**Table 5:** Multivariate adjusted odds ratios (95% CI) for abdominal obesity across quartile (Q) categories of dietary patterns scores.

	Animal Food Pattern Score			Traditional Chinese pattern Score			Western Fast-Food Pattern Score			High-Salt Pattern Score		
	Q1	Q4	*p*	Q1	Q4	*p*	Q1	Q4	*p*	Q1	Q4	*p*
Abdominal obesity												
Model 1	1.00	1.55 (1.144, 2.107)	0.005	1.00	0.68 (0.488, 0.936)	0.018	1.00	0.85 (0.628, 1.153)	0.297	1.00	1.03 (0.764, 1.382)	0.856
Model 2	1.00	1.67 (1.191, 2.345)	0.003	1.00	0.63 (0.438, 0.891)	0.009	1.00	0.88 (0.541, 1.143)	0.358	1.00	0.98 (0.705, 1.352)	0.886
Model 3	1.00	1.67 (1.188, 2.340)	0.003	1.00	0.63 (0.441, 0.901)	0.011	1.00	0.88 (0.625, 1.225)	0.437	1.00	0.94 (0.673, 1.320)	0.731

Model 1: adjusted for sex and age; Model 2: further adjusted for physical activity level; Model 3: additionally adjusted for total energy intake. Q4: the highest quartile of dietary patterns, Q1: the lowest quartile of dietary patterns; CI: confidence interval.

## 4. Discussion

In this cross-sectional study, we identified four dietary patterns: animal food, traditional Chinese, western fast-food, and high-salt patterns. Our findings suggested that the animal food pattern was positively associated with BMI and WC, and the traditional Chinese pattern was inversely associated with BMI and WC. However, the association of dietary patterns with BMI and WC was very weak. Moreover, further analysis indicated that the animal food pattern was associated with a higher risk of abdominal obesity, while the traditional Chinese pattern was associated with a lower risk of abdominal obesity. To the best of our knowledge, this is the first investigation from a middle-aged Chinese population to report the association of major dietary patterns with the indicators of obesity.

The animal food pattern was characterized by high intakes of rice, mushroom, red meat, fish and shrimp, seafood and fats/oils. In this study, we found a positive relationship between the animal food pattern and abdominal obesity. The results of this study were consistent with previous studies reporting a significant inverse association between food consumption in the western pattern and the risk of obesity [22,23,24]. The positive association between the animal food pattern and obesity may partly be attributable to this pattern**^’^**s unhealthy constituents (red meat and fats/oils). Red meat containing amounts of saturated fat and cholesterol, is considered an energy-dense food and the excess consumption may contribute to a surplus intake of energy, which may increase the risk of obesity [25]. In addition, higher intakes of meat were reported to be associated with weight gain in the European Prospective Investigation into Cancer and Nutrition-Potsdam Study [26]. Togo *et al*. also found that high consumption of meat is positively associated with BMI and WC [27]. Furthermore, in the present study, a higher intake of meat may reflect some undetected dietary behavior or lifestyle contributing to weight gain. Overall, these findings underscore the importance of animal food in the alarming prevalence of obesity in a Chinese population.

The traditional Chinese pattern, characterized by a high consumption of rice, steamed bun/noodles, coarse grains, tubers, fresh vegetables and fruits, fish and shrimp, miscellaneous beans and tea, is generally considered a healthy pattern. In the present study, we found an inverse relationship between this pattern and WC, BMI, and obesity. However, it should be noted that the association between this pattern and WC, and BMI is very weak. Our findings are consistent with previously reported findings in Chinese studies [28], suggesting that the “traditional” dietary pattern was negatively associated with weight gain in Chinese adults. The protective effect of the traditional Chinese pattern could be attributed to this pattern**^’^**s healthy constituents (e.g., whole grains, fresh vegetable and fruits, and beans) (Table S2). Whole grains and fresh vegetables and fruits contain large amounts of dietary fiber. Previous studies have found that the high consumption of dietary fiber is associated with a decreased risk of obesity [29]. In addition, some foods (e.g., whole grains and vegetables) in the traditional Chinese pattern have a low glycemic index (GI), which has been found to decrease the risk of obesity [30]. Kong *et al.* reported that low GI was associated with a decreased risk of obesity in the Chinese population [31]. However, a high glycemic index in the traditional Chinese pattern may be present because of a high rice intake. In this pattern, we found that rice had a high factor loading. Recently a study indicated that rice intake was inversely associated with weight gain [32]. Rice is a low-energy food, which may contribute to the buck of the traditional Chinese pattern. Compared with wheat flour, rice absorbs more water when cooked. Thus, the energy density of the rice is lower than the wheat staple diet. Previously some studies have indicated that high energy-dense diets are associated with an increased risk of obesity [33].

The western fast-food pattern was characterized by high intakes of fast foods, snacks, chocolates, coffee, and drinks. We did not find a significant association of this pattern with obesity in this study. The results are inconsistent with existing studies, which found that a high consumption of fast food was associated with the risk of overweight and obesity in the urban Chinese population [34]. The complex nature of this pattern may explain this finding to some extent. On the one hand, compared with the western dietary pattern (high consumption of refined grains, red meat, butter, high-fat dairy products, sweets and desserts, pizza and soft drinks) [23], the western fast-food pattern is characterized by high intakes of fast foods, snacks, chocolates, coffee, and drinks, and low intakes of red meat, butter, and high-fat dairy products in our study. Several studies have also suggested that the consumption of red meat is associated with an increased risk of obesity [35,36]. On the other hand, the study participants were predominately a group of Chinese population aged 45–60 years, who rarely consumed fast food, snacks, and drinks. In addition, epidemiological evidence and experimental studies have demonstrated that drinking tea is associated with a lower risk of obesity and related diseases [37,38]. Furthermore, no significant association could also be due to the reverse causality. Study participants with a risk of obesity might have been advised to reduce their fat intake, thereby changing dietary habits. This possibility cannot be excluded in a cross-sectional study.

The high-salt pattern was characterized by high intakes of pickled vegetables, processed and cooked meat, bacon, salted fish, and bean sauce in our analyses. Although a positive association of this pattern with BMI was found in women, the association was very weak. Moreover, we found no association of the high-salt pattern in relation to abdominal obesity. No significant association could be attributed to the complex constituents in this pattern. On the one hand, the unhealthy constituents (e.g., processed meat, cooked meat, and bacon) have been reported to be associated with a higher risk of obesity [39]. In addition, previous studies have shown that a high salt intake was associated with an increased risk of hypertension, an important risk factor for obesity [40]. On the other hand, some healthy foods such as vegetables and fish were also loaded in this pattern and could interact with other foods to counteract the adverse effect on obesity. As previously reported [29], high consumption of dietary fiber is associated with a decreased risk of obesity. Meanwhile, the omega-3 polyunsaturated fatty acid (omega-3PUFA) contained in fish has been reported to have a protective role against obesity [41]. Furthermore, physical activity as a form of energy expenditure, has been considered as a standard clinical recommendation for obese individuals [42]. In our analyses, participants in the highest quartile of the high-salt pattern have higher physical activity level than those in the lowest quartile. Some studies demonstrated that a higher levels of physical activity had a protective effect against long-term gain in weight [43,44].

## 5. Strengths and Limitations

The present study holds its strengths and limitations. Firstly, to our knowledge, this is the first study examining the associations between dietary patterns and the indicators of obesity among a middle-aged Chinese population. Because of unique diet cultures and backgrounds, our findings further identify the special dietary patterns of the middle-aged Chinese population. Secondly, the use of a validated semi-quantitative FFQ by a face-to-face interview ensured that the data we collected are accurate. Thirdly, we have adjusted for potential known confounders for reliability in the present study. Nevertheless, several potential limitations should be considered in this study. Firstly, given the cross-sectional design of this study, we are unable to assess the causal relationship between dietary patterns and obesity. Thus, our findings need to be confirmed in a future prospective study. Secondly, several subjective and arbitrary decisions in the use of factor analysis need to be considered [45]. Finally, the study participants are restricted to the middle-aged Chinese population in the city of Hangzhou, Zhejiang Province, east China. Thus, the conclusions may not be extrapolated to the entire Chinese population.

## 6. Conclusions

In conclusion, our findings suggested that the animal food pattern was associated with a higher risk of abdominal obesity, while the traditional Chinese pattern was associated with a lower risk of abdominal obesity among a Chinese population aged 45–60 years. Nevertheless, more prospective studies are required to clarify whether the causal associations exist between dietary patterns and the risk of obesity.

## Supplementary Files

Supplementary File 1
