# Supplementary material for: Association between Dietary Patterns and the Indicators of Obesity among Chinese: A Cross-Sectional Study
Source: Nutrients. 2015 Sep 17;7(9):7995–8009. doi: 10.3390/nu7095376 (PMC4586571; doi:10.3390/nu7095376)
Supplement: Supplementary File 1 [file nutrients-07-05376-s001.docx]

Supplementary Materials

**Table S1.** Food grouping used in the dietary pattern analyses.

| **Food groups** | **Food items** |
| --- | --- |
| Rice | Rice, porridge, rice in soup |
| Steamed bun/noodles | Steamed bun/noodles, instant noodles, wonton, dumplings, steamed stuffed bun |
| Coarse grains | Corn, sorghum, millet, oats |
| Tubers | Sweet potato, potato, taro |
| Fresh vegetables and fruits | Wild vegetables ,Green vegetable, spinach, green peppers, tomato Chinese cabbage, radish, cucumber, apple, peach, pear |
| Pickled vegetables | Salted vegetables, Chinese sauerkraut |
| Dried vegetable | Day lily, beans dry |
| Mushrooms | Mushroom, shiitakes, enoki |
| Seaweed and kelp | Seaweed and kelp |
| Red meat | Pork, mutton, beef |
| Poultry and organs | Chicken, duck, liver |
| Processed and cooked meat | Ham and sausage, sauced pork, roast duck |
| Fish and shrimp | Fish, shrimp |
| Eggs | Duck eggs, chicken eggs |
| Seafood | Fish, shrimp, crab ,squid, jellyfis, shellfish |
| Bacon and salted fish | Salted meat and duck, salted fish |
| Salted and preserved eggs | Salted duck and chicken eggs, preserved eggs |
| milk | Liquid milk, milk powder, yoghurt |
| Cheese | Cheese |
| Soybean and its products | Tofu, dried bean curd, soy milk |
| Miscellaneous bean | Mung beans, red beans, hemp beans |
| Bean sauce | Bean sauce |
| Fats/ oil | Soybean oil, tea oil, rapeseed oil, olive oil ,lard, butter |
| Fast foods | KFC, Mcdonald,fried dough sticks and twists, fried cakes |
| Nuts | Walnut, peanuts, almonds, melon seeds |
| Snacks | Cookies, sachima, bread, cake, ice cream, candy, sweets ,potato chips, shrimp roll, popcorn |
| Chocolates | Chocolates |
| Water | Water |
| Honey | Honey, hydromel |
| Drinks | Coca-cola, sprite, fruit and vegetable drink, fruits juice |
| Alcoholic beverages | Beer, fruit wine, grape wine |
| Tea | Tea,scented tea, wong Lo Kat |
| Coffee | Coffee |

**Table S2.** The key food intake of the study participants by quartile (Q) categories of dietary pattern scores.

|  | **Animal Food** | | **Traditional Chinese** | | **Western Fast-Food** | | **High-Salt** | |
| --- | --- | --- | --- | --- | --- | --- | --- | --- |
|  | **Q1 (*n* = 640)** | **Q4 (*n* = 640)** | **Q1 (*n* = 640)** | **Q4 (*n* = 640)** | **Q1 (*n* = 640)** | **Q4 (*n* = 640)** | **Q1 (*n* = 640)** | **Q4 (*n* = 640)** |
| Rice | 295.14 ± 164.89 | 349.17 ± 156.10 | 282.00 ± 149.89 | 365.76 ± 188.20 | 337.08 ± 191.82 | 294.55 ± 134.53 | 245.86 ± 140.65 | 282.69 ± 171.42 |
| Coarse grains | 20.90 ± 35.50 | 14.49 ± 23.77 | 19.28 ± 20.81 | 36.22 ± 35.10 | 18.43 ± 38.67 | 18.86 ± 27.32 | 22.18 ± 37.82 | 24.61 ± 23.72 |
| Tubers | 19.09 ± 33.79 | 20.06 ± 31.28 | 18.72 ± 17.49 | 28.88 ± 34.63 | 20.92 ± 38.20 | 16.92 ± 22.45 | 13.63 ± 19.45 | 19.78 ± 28.74 |
| Fresh vegetables and fruits | 440.32 ± 112.60 | 372.48 ± 125.70 | 327.31 ± 169.13 | 457.81 ± 149.59 | 388.68 ± 149.95 | 393.54 ± 286.24 | 377.73 ± 135.01 | 408.17 ± 166.97 |
| Red meat | 60.03 ± 31.61 | 74.48 ± 42.20 | 69.57 ± 39.18 | 65.46 ± 30.22 | 70.53 ± 24.90 | 87.88 ± 37.54 | 56.80 ± 34.91 | 67.93 ± 46.52 |
| Drinks | 10.69 ± 48.85 | 13.12 ± 18.14 | 17.16 ± 56.56 | 4.51 ± 20.78 | 10.69 ± 1.76 | 26.66 ± 21.54 | 10.00 ± 48.50 | 8.22 ± 33.83 |

Variables are presented as Mean ± standard deviation (SD); Q4: the highest quartile of dietary patterns, Q1: the lowest quartile of dietary.

© 2015 by the authors; licensee MDPI, Basel, Switzerland. This article is an open access article distributed under the terms and conditions of the Creative Commons Attribution license (http://creativecommons.org/licenses/by/4.0/).
